# General Consensus on Implementing an Episode-Based Payment Model: Results From the American College of Radiation Oncology (ACRO) Payment Reform Survey

**DOI:** 10.7759/cureus.81557

**Published:** 2025-04-01

**Authors:** Joseph A Wilding, James S Clifford, Dwight E Heron, Christopher D Jahraus, Jason McKitrick, Cecil M Benitez, Tarita O Thomas

**Affiliations:** 1 Department of Radiation Oncology, Virginia Commonwealth University, Richmond, USA; 2 Department of Public Health, East Carolina University, Greenville, USA; 3 Department of Radiation Oncology, Bon Secours Mercy Health System, Youngstown, USA; 4 Department of Radiation Oncology, Generations Radiotherapy & Oncology PC, Alabaster, USA; 5 Department of Health Policy, Liberty Partners Group, Washington, D.C., USA; 6 Department of Radiation Oncology, University of California Los Angeles (UCLA), Los Angeles, USA; 7 Department of Radiation Oncology, Northwestern University Feinberg School of Medicine, Chicago, USA

**Keywords:** cms, episode-based payment, health policy, medicare, oncology, payment reform, radiation oncology, radiation oncology case rate, rocr, value-based care

## Abstract

Introduction

Medicare payments for radiation oncology (RO) services adhere to a fee-for-service model. However, a growing number of stakeholders, including both payers and physicians, are increasingly supportive of transitioning to an episode-based payment (EBP) model. Multiple novel models have been proposed, including the Centers for Medicare and Medicaid Services/Center for Medicare & Medicaid Innovation Radiation Oncology Model and the American Society for Radiation Oncology Radiation Oncology Case Rate (ROCR) program. Data regarding the level of RO physician support for implementing an EBP model, and RO physician opinions on key elements of the proposed models, have not been published. The American College of Radiation Oncology (ACRO) Government Relations and Economics Committee conducted a robust survey to gain insight into physician sentiment, enhance transparency, promote dialogue, and assess consensus on payment reform.

Methods

A 29-item questionnaire was created and distributed electronically via email to practicing RO attending and resident physicians, identified using American Medical Association Physician Professional Data and ACRO membership rolls. The survey commenced on October 2, 2023, and concluded on February 1, 2024. Hypothesis testing was conducted using the null hypothesis that there is no consensus amongst physicians on a given item (i.e., the proportion of physicians agreeing or disagreeing with a statement defined as equal to 50%). One-sample tests of proportions, specifying the null hypothesis as 0.5, were run using R 4.3.3 (R Core Team, Vienna, Austria), a statistical computing software.

Results

A total of 528 responses were collected, of which 500 were from practicing RO physicians in the United States (U.S.) and were included for analysis. Respondents included attending physicians from all 50 U.S. states, the District of Columbia, and Puerto Rico. It was found that 61.0% (n = 285 of 467; p < 0.001; 95% CI: 56.4%-65.4%) of respondents support implementing an EBP model for RO services, in which payment is primarily based on the site of disease being treated, rather than the X-ray beam modality or fraction number; 17.3% (n = 81 of 467) neither support nor oppose it, and 21.6% (n = 101 of 467) oppose such a model. Support for EBP exceeds 50.0% across all experience levels, practice types (academic, community or private practice, and Veteran’s Health Administration), practice sites (hospital and freestanding), practice settings (rural, suburban, and urban), and U.S. geographic regions. It was found that 63.8% (n = 298 of 467) of respondents agree that such a model would better align financial incentives with clinical guidelines. Additionally, 78.6% (n = 367 of 467) support site-neutral payments that equalize pay for RO services, regardless of whether treatments are delivered at a freestanding radiation therapy center or hospital outpatient department. Although not directly tied to EBP models, 68.7% (n = 321 of 467) support site-neutral direct supervision requirements, with specified limited exceptions.

Conclusion

A clear majority of RO physicians currently practicing in the U.S. either support or are neutral towards the implementation of an EBP model for their specialty. This survey represents the first comprehensive assessment of practicing RO physicians’ views on implementing an EBP model. The findings provide critical insight for RO stakeholders, including members of Congress, considering the ROCR program.

## Introduction

National Health Expenditures (NHE) in the United States (U.S.) as a percentage of gross domestic product (GDP) increased from 5.0% in 1960 to 17.2% in 2010. It peaked at 19.5% in 2020 amid the COVID-19 pandemic. In 2023, it was $4.9 trillion, representing 17.6% of GDP (Centers for Medicare and Medicaid Services (CMS) NHE Accounts; NHE Summary, including share of GDP, CY 1960-2023). From 2000 to 2010, office-based radiation oncology (RO) patient volume increased by 8.2%, while RO Medicare Part B payments increased by 217%, which led to interest in cutting costs in RO [1]. Such cuts did occur, as evidenced by a greater than 21% decrease in relative value units (RVUs) for RO since 2006 [2]. In this context of instability, value-based care models that incentivize cost-effectiveness, specifically episode-based payment (EBP) models, have gained interest among various RO stakeholders.

The largest single-payer for healthcare services in the U.S. is the CMS. Medicare is a federal health insurance program for patients aged 65 and older, as well as younger people with certain long-term disabilities. Medicare expenditures, including both Original Medicare (Traditional Medicare) and Medicare Advantage, accounted for 20.9% of NHE in 2022, with $944.3 billion spent on 65.1 million enrollees [1,3]. Traditional Medicare has historically operated under a fee-for-service (FFS) model, with outpatient medical care covered under Medicare Part B. In 2022, Traditional Medicare Part B paid $208.6 billion, serving 33.8 million people [3]. Medicare pays different rates for otherwise identical radiation treatment, depending on the setting where treatments occur, with payments to hospital outpatient departments significantly exceeding those made to freestanding medical offices not affiliated with hospitals [4].

Enacted on December 28, 2015, the Patient Access and Medicare Protection Act froze payment rates for key RO services and required the Secretary of Health and Human Services (HHS) to report to Congress on the development of an episodic alternative payment model (APM) for certain Medicare radiation therapy coverage [5]. HHS submitted its report on November 3, 2017, and, on July 10, 2019, the CMS Center for Medicare & Medicaid Innovation proposed the RO model or RO-APM [6,7]. The RO-APM would have shifted payments from an FFS model to an EBP model for certain RO services. It would have been mandatory for a substantial portion of practices in certain locations, accounting for approximately 30% of all RO episodes, starting on January 1, 2021. Prospective Payment System-Exempt Cancer Hospitals (PPS-Exempt Cancer Hospitals or PCHs), a group of 11 cancer hospitals that receive cost-based reimbursement from CMS rather than payment through the standard PPS, were to be excluded from the RO-APM [8].

Implementation of the RO-APM was delayed multiple times, in part due to concerns that the model could threaten the financial viability of certain practices, particularly freestanding centers and those serving underserved areas. It is known that geographic adjustments to payments have benefited practices in urban areas more than those in rural areas [9]. Further, there is a mismatch between the geographic location of attending RO physicians and Medicare beneficiaries, which disproportionately affects nonmetropolitan communities with a higher percentage of Black non-Hispanic constituents [10]. The Consolidated Appropriations Act, 2021, delayed implementation to January 1, 2022. The Protecting Medicare and American Farmers from Sequester Cuts Act then further delayed implementation to January 1, 2023. On August 29, 2022, CMS indefinitely deferred the start date of the RO-APM to “a date to be determined through future rulemaking” [11].

On June 28, 2023, the American Society for Radiation Oncology (ASTRO) announced it would pursue legislation to create a Radiation Oncology Case Rate (ROCR) payment program under Traditional Medicare. On May 14, 2024, the ROCR Value-Based Program Act of 2024 (S.4330) was introduced to the Senate, and a companion bill was introduced in the House of Representatives the following day (H.R.8404) [12,13]. Though they had bipartisan support, those bills did not pass the 118th Congress. Updated ROCR bills were reintroduced to the 119th Congress, in the Senate on March 13, 2025 (S.1031), and in the House of Representatives the following day (H.R.2120) [14,15]. Like the RO-APM, ROCR is an EBP model.

If enacted into law, ROCR will be mandatory for almost all outpatient RO services, though PPS-Exempt Cancer Hospitals will be excluded from the model. Brachytherapy, radiopharmaceuticals, and proton beam therapy (PBT) services will also be excluded. Payments will be site-neutral but adjusted based on practice accreditation status. To address health disparities, add-on payments will be made to cover transportation services for patients with transportation insecurity. ROCR is intended to reduce annual Medicare spending on RO services by roughly 1% from current levels, and then maintain sustainable, inflation-adjusted reimbursement levels into future years [16].

Given these proposed legislative changes, this study was conducted to examine RO physician support for implementing an EBP model and opinions on elements of proposed models. It was hypothesized that consensus support for implementing EBP exists, but elements of proposed models may lack consensus support or even have consensus opposition. We developed a comprehensive, confidential survey to garner a deeper understanding of these issues. To the best of our knowledge, this work represents the first of its kind survey across all practice environments and all levels of experience to ascertain how RO physicians view payment reform in the U.S.

Preliminary results of this survey were presented at the Radiation Oncology Summit: ACRO 2024, on March 14, 2024.

## Materials and methods

Study population

Data for this project were collected between October 2, 2023, and February 1, 2024. This study aimed to survey the total population of practicing RO attending and resident physicians in the U.S.; potential participants were identified using American Medical Association (AMA) Physician Professional Data (6,277 individuals) and American College of Radiation Oncology (ACRO) active membership rolls (1,167 individuals). The AMA and ACRO datasets were combined to develop a composite list of unique individuals and associated email addresses (6,385 email addresses) to which the survey was distributed. Practicing RO physicians whose current email addresses were not identified in the datasets were given the option to use a separate online form to request that the survey be sent to their preferred email address. Such requests were individually verified to ensure no respondent completed the survey multiple times.

Survey construction

Initially, a 27-item questionnaire was constructed to assess the degree of consensus on key aspects of proposed RO EBP models. The questionnaire was novel, with no prior testing or validation. The questionnaire was revised twice following its initial distribution. The first revision occurred after 69 responses were collected and involved adding a question about the respondent's practice setting (i.e., urban, suburban, and rural). The second revision took place after 159 responses were collected and added a question about practice accreditation status. The survey can be broken down into two distinct sections: demographics and opinions.

Demographics

Respondents were asked about their experience level, practice size, practice type (e.g., academic, community, or private practice), practice site (e.g., freestanding and hospital), geographic location, and whether they practice at one of the 11 PPS-Exempt Cancer Hospitals. Participants were asked if their practice used specific radiation delivery methods, including brachytherapy, adaptive radiotherapy (ART), radiopharmaceuticals, and PBT. The survey also inquired about physicians’ weekly work hours and compensation structure.

Opinions

Respondent support or opposition toward potential future RO-specific payment model reform was assessed. Opinions on an EBP model based on disease site, rather than on X-ray beam treatment modality (e.g., 3D conformal, intensity-modulated, and stereotactic), or fraction number, were assessed. Views toward potential elements of payment models were explored, including site neutrality, inflationary adjustments, adjustments based on accreditation status, and payments for transportation services. Potential exclusions to an EBP model were assessed, including brachytherapy, ART, radiopharmaceuticals, and PBT. The potential impacts of an EBP model were explored, including alignment of financial incentives with clinical guidelines, simplification of billing requirements, and payment stability and predictability. Respondent confidence in answering questions about Medicare payment models, opinions toward supervision requirements, and willingness to reduce Medicare payments in exchange for implementation of an EBP model were also assessed. All variables were measured on a five-point Likert scale, anchored with “Strongly Disagree” (1) and “Strongly Agree” (5). Each item was collapsed into a three-level ordinal variable, with “Strongly Agree” and “Agree” recoded as 1, “Strongly Disagree” and “Disagree” recoded as 2, and “Neither agree nor disagree” recoded as 3.

Survey instrument

The survey questions are available for review in Appendix 1. Invitations to complete the questionnaire were distributed via email through the survey platform SurveyMonkey™ (Momentive, San Mateo, CA, USA). Unique hyperlinks were sent to each potential participant, permitting only a single response per recipient to enhance result integrity. Personally identifiable information was omitted from the data collection process to preserve respondent anonymity. A total of seven reminder emails were sent at variable intervals throughout the 123-day collection period to individuals who had not completed the survey. Participation was promoted via word-of-mouth, social media posts, and direct email campaigns.

**Figure 1:**
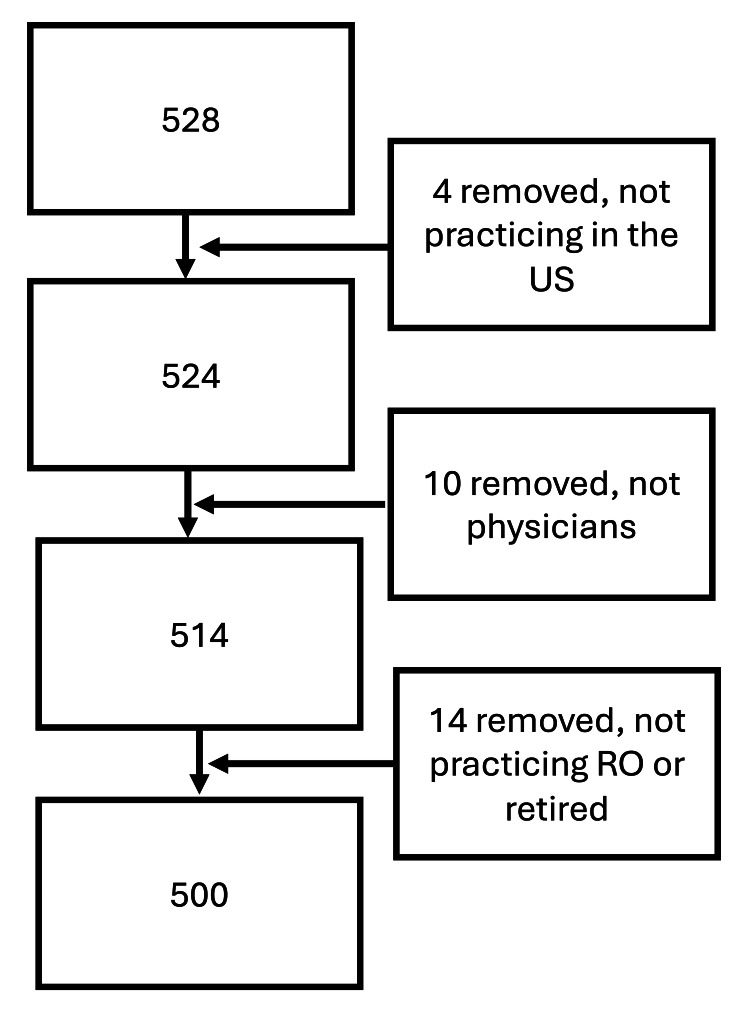
Flowchart of Participants RO, Radiation Oncology

A total of 528 individuals responded to the survey. Many of the survey’s demographic questions provided respondents with an opportunity to report if they were not physicians currently practicing RO. Primary practice location was also asked. Data were restricted to active physicians who were practicing RO in the U.S. for these analyses (n = 500; Figure 1).

Statistical analyses

All variables were summarized with frequency counts and percentages, as outlined in Table 1. Hypothesis tests were all conducted using the following null and alternative hypotheses: (1) the null hypothesis (H0) is that there is no consensus among physicians on a given item (i.e., the proportion of physicians agreeing or disagreeing with a statement is equal to 50%), while (2) the alternative hypothesis (HA) is that there is consensus among physicians for a given item (i.e., the proportion of physicians agreeing or disagreeing with a statement is not equal to 50%). One-sample tests of proportions, specifying the null hypothesis as 0.5, were run using R 4.3.3 (R Core Team, Vienna, Austria), a statistical computing software.

**Table 1:** Participant Characteristics ^1^Early-Career defined as 10 years or less out of training, Mid-Career as 11-20 years out of training, Late-Career as 21 or more years out of training; ^2^Number of practicing Radiation Oncologists; ^3^Veteran’s Health Administration

	n	%
Experience Level^1^		
Resident	52	10.4
Early-Career	156	31.2
Mid-Career	126	25.2
Late-Career	166	33.2
Practice Size^2^		
1	53	10.6
2-5	168	33.6
6-10	103	20.6
11-19	94	18.8
≥20	82	16.4
Practice Type		
Academic	189	37.8
Community or Private	306	61.2
VHA^3^	5	1.0
Practice Site		
Hospital	340	68.0
Freestanding	160	32.0
Practice Setting		
Rural	59	13.6
Suburban	153	35.3
Urban	221	51.1
U.S. Region		
Northeast	88	17.6
South	196	39.3
Midwest	126	25.3
West	86	17.2
Puerto Rico	3	0.6

## Results

Demographics

Participant Characteristics

**Figure 2:**
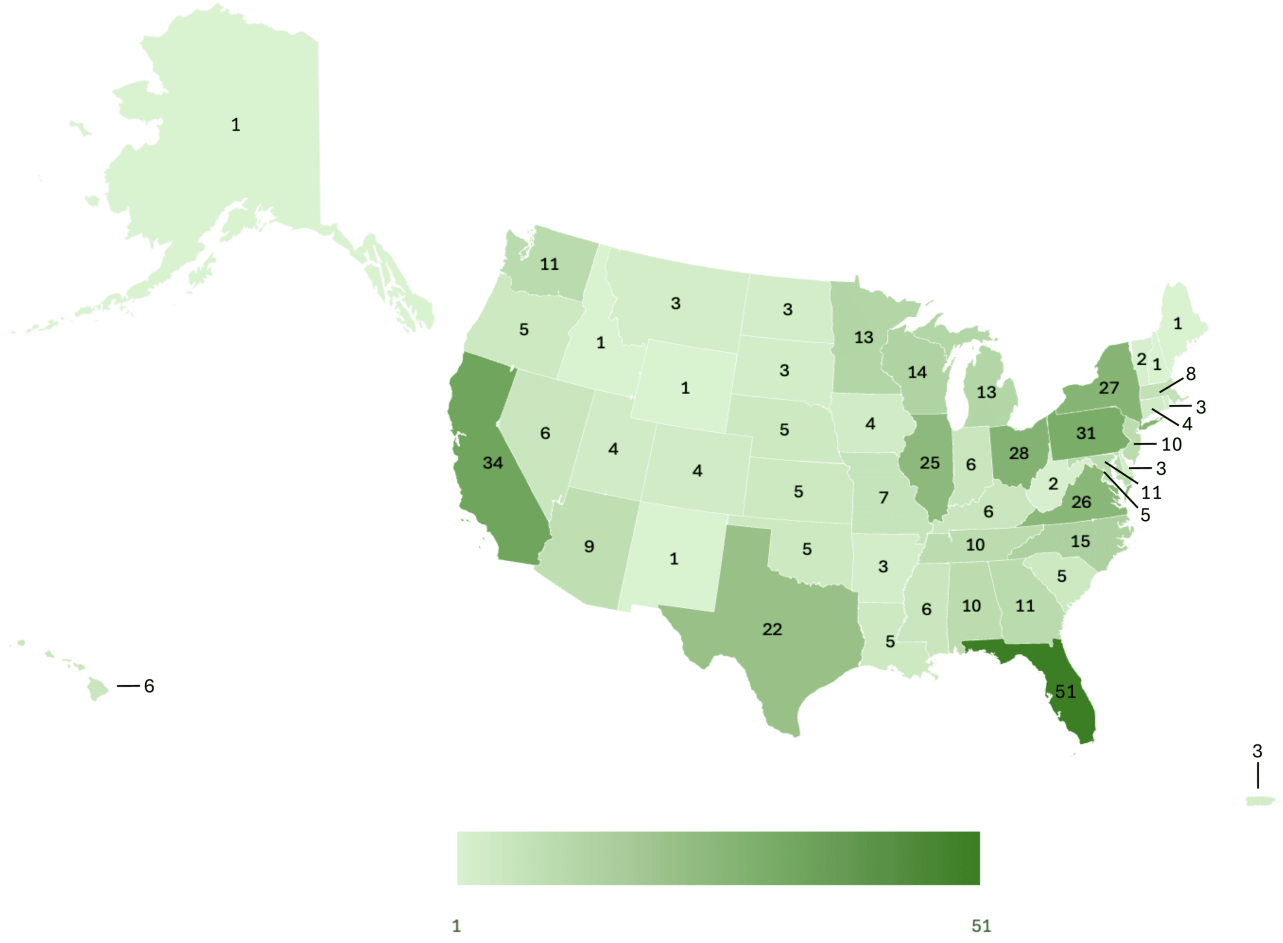
Geographic Distribution of Survey Participants

A majority of the 500 included respondents were attending RO physicians (n = 448 of 500; 89.6%), had 21 years or more of experience (n = 166 of 500; 33.2%) or 10 years or less out of training (n = 159 of 500; 31.2%), were in practices with 2-5 radiation oncologists (n = 168 of 500; 33.6%) or 6-10 radiation oncologists (n = 103 of 500; 20.6%), were in the community or private practice (n = 306 of 500; 61.2%), practiced at a hospital (n = 340 of 500; 68.0%), and were not at PPS-Exempt Cancer Hospitals (n = 464 of 500; 92.8%). Most respondents practiced in the South (n = 196 of 499; 39.3%) or the Midwest (n = 126 of 499; 25.3%), as defined by the U.S. Census Bureau. Responses were collected from attending physicians in all 50 states, the District of Columbia, and Puerto Rico. For further details on location data, see Figure 2. Of those respondents who provided data on practice setting, a majority practiced in an urban setting (n = 221 of 433; 51.0%).

Work Hours

Median weekly work hours for both attending and resident physicians were 50-59. More attendings (n = 209 of 448; 46.7%) than residents (n = 13 of 52; 25.0%) worked 49 or fewer hours. Fewer attendings (n = 83 of 448; 18.5%) than residents (n = 18 of 52; 34.6%) who worked 60 or more hours.

Compensation

All 448 attending RO physicians provided information on their compensation structure. Of those, 19 reported working locum tenens positions. Further analysis was performed on the remaining 429 respondents. A majority were paid a predetermined base salary (n = 224 of 429; 52.2%). Of those, nearly half were eligible for a bonus for exceeding target work relative value units (wRVUs) (n = 111 of 224; 49.6%), and more than one-third reported a component of their base salary was allocated for administrative tasks (n = 81 of 224; 36.2%).

There were differences in practice type and likelihood of being paid a predetermined base salary. A minority of RO physicians in the community or private practice received a predetermined base salary (n = 116 of 290; 40.0%), but a majority of those in academics (n = 103 of 134; 76.9%), and all those at the VHA (n = 5 of 5; 100.0%) did. A minority of attending physicians received professional component (PC) payments (n = 179 of 429; 41.7%). Of those, a minority also received technical component (TC) payments (n = 62 of 179; 34.6%).

Opinions

Confidence

“I feel confident in my ability to answer questions about current and proposed Medicare payment models for radiation oncologists.”

A majority of participants (n = 248 of 467; 53.1%) felt confident answering questions about current and proposed Medicare payment models. There were differences in practice type and likelihood of feeling confident. A majority of those in the community or private practice felt confident (n = 173 of 287; 60.3%), but only a minority of those in academics (n = 73 of 175; 41.7%) or at the VHA (n = 2 of 5; 40.0%) did.

There were also differences in experience level and likelihood of feeling confident. A minority of resident physicians felt confident (n = 16 of 46; 34.8%), while a plurality of early-career physicians did (n = 70 of 150; 46.7%). A majority of mid-career physicians (n = 75 of 120; 62.5%) and late-career physicians (n = 87 of 151; 57.6%) felt confident.

Episode-Based Payments (EBP)

“I support the implementation of episode-based payments for radiation oncology services in which the payment amount is primarily based on the site of disease being treated and not on the X-ray beam modality or fraction number.”

A majority support the implementation of EBP (n = 285 of 467; 61.0%; p < 0.001, 95% CI: 56.4%-65.4%). Some neither support nor oppose EBP (n = 81 of 467; 17.3%), or oppose EBP (n = 101 of 467; 21.6%).

**Figure 3:**
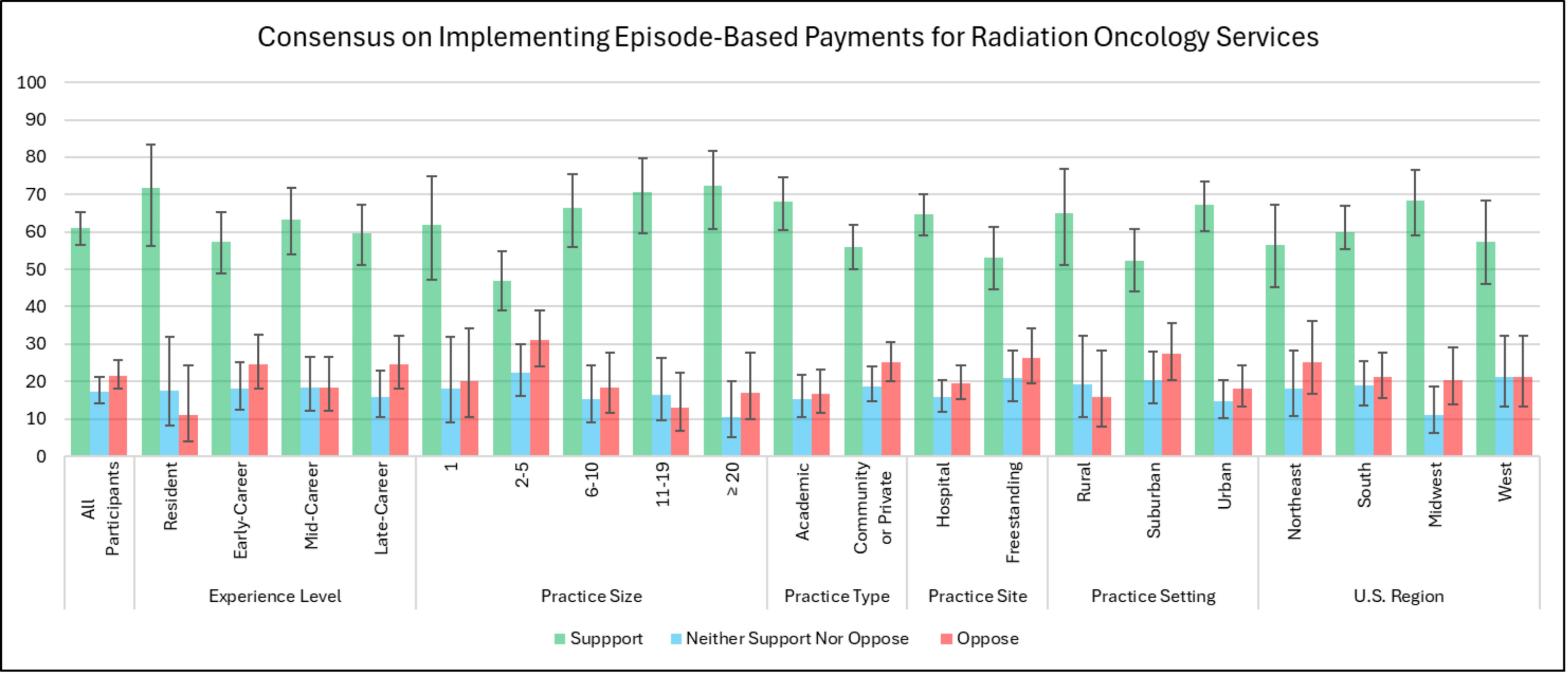
Consensus on Implementing Episode-Based Payments for Radiation Oncology Services Error bars denote 95% confidence intervals.

There is consensus support of greater than 50.0% across all experience levels, practice types, practice sites, practice settings, and U.S. geographic regions for disease site-based EBP. There is consensus support for practices with one radiation oncologist and for practices with six or more radiation oncologists (Figure 3 and Table 2).

**Table 2:** Consensus on Implementing Episode-Based Payments for Radiation Oncology Services ^1^Early-Career defined as 10 years or less out of training, Mid-Career as 11-20 years out of training, Late-Career as 21 or more years out of training; ^2^Number of practicing Radiation Oncologists; ^3^Veteran’s Health Administration (VHA)

	Support	Neutral	Oppose
Experience Level^1^			
Resident (n = 46)	33 (71.7%)	8 (17.4%)	5 (10.9%)
Early-Career (n = 150)	86 (57.3%)	27 (18.0%)	37 (24.7%)
Mid-Career (n = 120)	76 (63.3%)	22 (18.3%)	22 (18.3%)
Late-Career (n = 151)	90 (59.6%)	24 (15.9%)	37 (24.5%)
Practice Size^2^			
1 (n = 50)	31 (62.0%)	9 (18.0%)	10 (20.0%)
2-5 (n = 158)	74 (46.8%)	35 (22.2%)	49 (31.0%)
6-10 (n = 98)	65 (66.3%)	15 (15.3%)	18 (18.4%)
11-19 (n = 85)	60 (70.6%)	14 (16.5%)	11 (12.9%)
≥20 (n = 76)	55 (72.4%)	8 (10.5%)	13 (17.1%)
Practice Type			
Academic (n = 175)	119 (68.0%)	27 (15.4%)	29 (16.6%)
Community or Private (n = 287)	161 (56.1%)	54 (18.8%)	72 (25.1%)
VHA^3^ (n = 5)	5 (100.0%)	0 (0.0%)	0 (0.0%)
Practice Site			
Hospital (n = 318)	206 (64.8%)	50 (15.7%)	62 (19.5%)
Freestanding (n = 149)	79 (53.0%)	31 (20.8%)	39 (26.2%)
Practice Setting			
Rural (n = 57)	37 (64.9%)	11 (19.3%)	9 (15.8%)
Suburban (n = 143)	75 (52.4%)	29 (20.3%)	39 (27.3%)
Urban (n = 204)	137 (67.2%)	30 (14.7%)	37 (18.1%)
U.S. Region			
Northeast (n = 83)	47 (56.6%)	15 (18.1%)	21 (25.3%)
South (n = 185)	111 (60.0%)	35 (18.9%)	39 (21.1%)
Midwest (n = 117)	80 (68.4%)	13 (11.1%)	24 (20.5%)
West (n = 80)	46 (57.5%)	17 (21.3%)	17 (21.3%)
Puerto Rico (n = 1)	1 (100.0%)	0 (0.0%)	0 (0.0%)

Expected Impact of EBP

“Episode-based payments for radiation oncology services in which the payment amount is primarily based on the site of disease being treated and not on the X-ray beam modality or fraction number would result in…”

“better alignment of financial incentives with clinical guidelines”

A majority agree with this statement (n = 298 of 467; 63.8%). Some neither agree nor disagree (n = 84 of 467; 18.0%), or disagree (n = 85 of 467; 18.2%).

“more stable and predictable payments”

A majority agree with this statement (n = 287 of 467; 61.5%). Some neither agree nor disagree (n = 95 of 467; 20.3%) or disagree (n = 85 of 467; 18.2%).

“more simplified billing”

A majority agree with this statement (n = 331 of 467; 70.9%). Some neither agree nor disagree (n = 77 of 467; 16.5%) or disagree (n = 59 of 467; 12.6%).

Exclusions

“If episode-based payments for radiation oncology services are implemented, “…” should be excluded from that model and continue to be paid for through the current fee-for-service system.”

“…brachytherapy…”

A majority of participants’ practices treat patients with brachytherapy (n = 416 of 500; 83.2%), and a majority of participants support excluding brachytherapy from an EBP model (n = 285 of 467; 61.0%). Some neither support nor oppose this exclusion (n = 82 of 467; 17.6%), or oppose it (n = 100 of 467; 21.4%).

“…radiopharmaceuticals…”

A majority of participants’ practices treat patients with radiopharmaceuticals (n = 282 of 500; 56.4%), and a majority of participants support excluding radiopharmaceuticals from a potential EBP model (n = 251 of 467; 53.7%). Some neither support nor oppose this exclusion (n = 137 of 467; 29.3%) or oppose it (n = 79 of 467; 16.9%).

“…real-time adaptive radiotherapy…”

A minority of participants’ practices treat patients with real-time ART (n = 156 of 500; 31.2%). A plurality of participants support excluding real-time ART from a potential EBP model (n = 200 of 467; 42.8%). Others neither support nor oppose this exclusion (n = 126 of 467; 27.0%) or oppose it (n = 141 of 467; 30.2%).

“…proton beam therapy…”

A minority of participants’ practices treat patients with PBT (n = 91 of 500; 18.2%), and a minority of participants support excluding PBT from a potential EBP model (n = 110 of 467; 23.6%). Some neither support nor oppose this exclusion (n = 110 of 467; 23.6%). A majority oppose it (n = 247 of 467; 52.9%).

Amongst participants whose practices do treat patients with PBT, a plurality support excluding PBT from a potential EBP model (n = 41 of 88; 46.6%). Some neither support nor oppose this exclusion (n = 17 of 88; 19.3%) or oppose it (n = 30 of 88; 34.1%).

Among participants whose practices do not treat patients with PBT, a minority support excluding PBT from a potential EBP model (n = 69 of 379; 18.2%). Some neither support nor oppose this exclusion (n = 93 of 379; 24.5%). A majority oppose it (n = 217 of 379; 57.3%).

Reducing Medicare Payments by 1% in Exchange for EBP

“If most radiation oncologists support a specific episode-based payment model, then I would be willing to support a 1% reduction in Medicare payments for radiation oncology services in exchange for federal legislation that implements that model.”

A minority of participants support reducing Medicare payments by 1% in exchange for EBP legislation (n = 175 of 467; 37.5%). Some neither support nor oppose that exchange (n = 99 of 467; 21.2%), while a plurality oppose it (n = 193 of 467; 41.3%).

Site-Neutrality Payments

“I support site-neutral payments that equalize compensation for radiation oncology services regardless of whether treatments are delivered at a freestanding radiation therapy center or hospital outpatient department.”

A majority support site-neutrality (n = 367 of 467; 78.6%). Some neither support nor oppose site-neutral payments (n = 58 of 467; 12.4%), while others oppose them (n = 42 of 467; 9.0%).

Accreditation

“I support adjusting radiation oncology payments based on whether a practice is accredited by a recognized credentialing body.”

A majority of participants’ practices were accredited by a national credentialing body (n = 266 of 346; 76.9%). Some were either not accredited (n = 56 of 346; 16.2%) or were unsure of their practice’s accreditation status (n = 24 of 346; 6.9%). A majority support adjusting payments based on accreditation status (n = 256 of 467; 54.8%). Some neither support nor oppose that adjustment (n = 85 of 467; 18.2%), or oppose it (n = 126 of 467; 27.0%).

Among only participants whose practices are not accredited, a minority support adjusting payments based on accreditation status (n = 11 of 52; 21.2%). Some neither support nor oppose this adjustment (n = 10 of 52; 19.2%). A majority oppose it (n = 31 of 52; 59.6%).

Transportation Payments

“I support paying radiation oncology practices for providing transportation services to patients who do not have reliable transportation to their treatments.”

A majority of participants support payments for providing transportation services (n = 405 of 467; 86.7%). Some neither support nor oppose such payments (n = 49 of 467; 10.5%), or oppose them (n = 13 of 467; 2.8%).

Inflationary Adjustments

“I support annual adjustments to radiation oncology payments to account for inflation.”

A vast majority support annual inflationary adjustments (n = 448 of 467; 95.9%). Still, some neither support nor oppose annual inflationary adjustments (n = 10 of 467; 2.1%), or oppose them (n = 9 of 467; 1.9%).

Supervision Requirements

“I support site-neutral direct supervision requirements with limited exceptions (e.g., allow for tumor board attendance, allow for completion of emergency department and inpatient consultations).”

A majority of participants support site-neutral direct supervision requirements with limited exceptions (n = 321 of 467; 68.7%). Some neither support nor oppose these supervision requirements (n = 73 of 467; 15.6%), or oppose them (n = 73 of 467; 15.6%).

## Discussion

This survey represents the first comprehensive assessment of practicing RO physicians’ views on the implementation of an EBP model for RO services, and it has revealed physicians’ broad consensus support for implementing payment reform. Participants represented a wide cross-section of the specialty, including attending physicians from all 50 U.S. states, the District of Columbia, and Puerto Rico. Greater than 50.0% support for implementing an EBP model was found across all experience levels (resident, early-career, mid-career, and late-career), practice types (academic, community or private practice, and Veteran’s Health Administration), practice sites (hospital and freestanding), practice settings (rural, suburban, and urban), and U.S. geographic regions (Northeast, South, Midwest, and West). These findings bring to light a widespread consensus among U.S. RO physicians, with 78.3% either in favor of or neutral towards transitioning from the traditional FFS model to an EBP model, specifically one that aligns payments primarily with the site of disease rather than the modality of treatment or fraction number. This further reflects a growing recognition within the specialty that payment structures need to evolve to better incorporate value-based care principles.

One of the key takeaways from the survey is the high level of support for site-neutral payments, with 78.6% of respondents supporting equalizing payments between hospital outpatient departments and freestanding centers. Rasmussen et al. demonstrated that in practices with a high cost of equipment, there is a substantial decrement in reimbursement in freestanding centers compared to the hospital setting [2]. The strong support for site-neutrality suggests that RO physicians recognize the need for a more equitable and standardized payment approach across different practice environments. This is significant because site-neutrality has been a core component of many proposed payment reform efforts, including ROCR (S.1031 and H.R.2120), a bipartisan initiative that, if passed into law, would equalize payment rates between different treatment settings through an EBP model [14,15].

The survey also underscores the potential of an EBP model to better align financial incentives with clinical guidelines, as 63.8% of respondents agreed that an EBP model would achieve this goal. Such alignment is essential to ensure that guideline-directed therapy is rewarded rather than penalized. Additionally, 61.5% of respondents indicated that an EBP model would result in more stable and predictable payments, potentially increasing confidence to invest in high-cost equipment upgrades that can enhance patient care. Furthermore, 70.9% of respondents agreed that an EBP model would simplify billing, which may reduce administrative burdens and improve the efficiency of RO practices.

Despite the overall support for EBP models, the survey reveals some areas of concern that warrant further consideration. Notably, there is consensus opposition to excluding PBT from an EBP model, with 52.9% opposing its exclusion. This opposition was greatest among physicians whose practices do not treat patients with PBT, with 57.3% opposing its exclusion. However, even among participants whose practices do treat patients with PBT, there was no consensus on whether PBT should be excluded, with only 46.6% supporting its exclusion.

**Figure 4:**
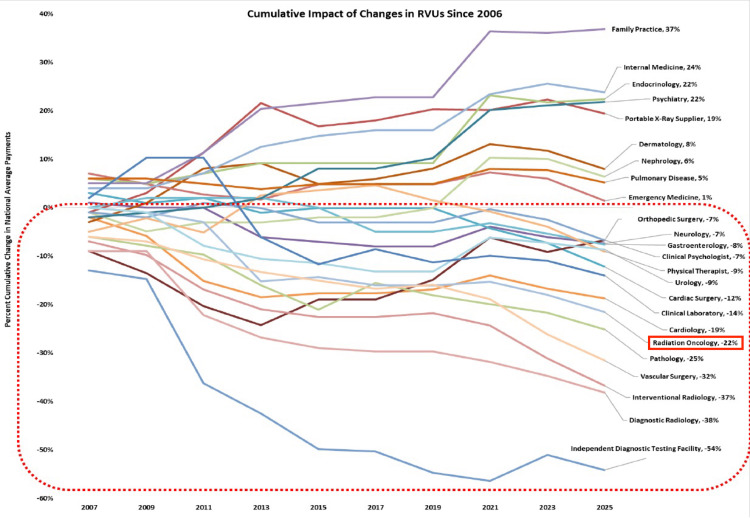
Cumulative Impact of Changes in RVUs Since 2006 Source: HMA analysis 2007-2025 Medicare Physician Fee Schedule Impact Tables. Permission to publish this image was obtained from HMA. The values presented for 2021-2025 are adjusted to reflect the effects of the CAA, 2021, 2022, 2023, and 2024, and the expected conversion factor reduction in 2025. The cumulative impact of changes reflects averages at the specialty level and does not account for changes in volume, mix or intensity of services, or site of service changes over the same timeframe. The cumulative impact at an individual practitioner or practice level may be different from what is presented. The inclusion of this figure in ACRO-related publications does not reflect HMA endorsement of the article content presented (December 2, 2024). This figure was initially prepared by HMA for the Office-Based Facility Association, of which ACRO is a member. A prior version, based on the 2025 Physician Fee Schedule (PFS) proposed rule, was previously published by Rasmussen et al. [2]. This version has been updated based on the 2025 PFS final rule. HMA, Healthcare Management Associates, ACRO, American College of Radiation Oncology; CAA, Consolidated Appropriations Act; RVUs, Relative Value Units

The willingness of only 37.5% of respondents to support a 1% reduction in Medicare payments in exchange for the implementation of an EBP model indicates that, despite widespread support for an EBP model, there is strong concern about even modest additional cuts to current Medicare payments. This is understandable, as Rasmussen et al. found that, in the calendar year 2024, CMS payments for 195 procedures performed in an office (freestanding) setting were less than the direct costs associated with those procedures, as calculated by CMS itself. This included many radiotherapy codes in the Medicare Physician Fee Schedule [2]. Further, changes in RVUs have resulted in a 22% decrease in payments for RO services since 2006, as shown in Figure 4. Any future payment model reforms will need to carefully balance the goal of cost reduction with the need to maintain viable and sustainable reimbursement for RO services.

Though not directly tied to EBP models, it is also notable that 68.7% of participants support site-neutral direct supervision requirements with limited exceptions. This demonstrates consensus on the importance of RO physicians being physically present and immediately available to render assistance as needed during radiation treatments across different care settings, with reasonable exceptions. With consideration given to member feedback and preliminary results of this survey, on May 3, 2024, ACRO issued a Statement on Radiation Therapy Supervision, endorsing “direct supervision in all sites of service at the initiation of radiation therapy, which may then be followed by general supervision at the discretion of the Radiation Oncologist” [17].

This study has inherent attributes related to its cross-sectional design and reliance on self-reported data. While this design enabled the collection of relevant data in a timely and efficient manner, it could not capture longitudinal changes in opinions toward payment reform over time. Additionally, the survey solicited participants’ opinions on EBP models generally, rather than focusing on any specific proposed models, such as ROCR or specific aspects of that model. As a result, while the findings offer broad applicability, they do not directly indicate levels of support or opposition to any one particular model.

It is important to note that this survey was revised on two occasions following its initial distribution to include additional questions regarding respondents’ practice settings and practice accreditation status. Another limitation stems from the survey's treatment of ART. While participants were asked about their practices’ use of real-time ART and their opinions on its exclusion from an EBP model, the introductory section of the survey defined real-time ART using terminology more commonly associated with online ART. This discrepancy may have led to variability in interpretation and responses.

The most notable strength of this study is the robust and diverse participation of practicing RO physicians from across the U.S., including all 50 states, the District of Columbia, and Puerto Rico. The survey captures responses from physicians spanning a wide spectrum of experience levels, practice types, practice sites, and practice settings. This comprehensive representation significantly enhances the generalizability of the findings, ensuring that the breadth and diversity of the field are reflected.

## Conclusions

This survey provides valuable insights into the perspectives of U.S. RO physicians regarding payment reform, particularly the adoption of an EBP model. The findings demonstrate broad support for a site-neutral model in which payments for radiotherapy treatments are primarily based on the disease site being treated, not on the specific modality used or the number of fractions. However, the survey also highlights areas of concern, including the exclusion of PBT and the potential financial impact of reduced Medicare payments.

As the field of RO continues to evolve, these insights will be crucial for informing the development and implementation of payment models that balance cost-effectiveness with the need to provide high-quality patient care. The robust response to this survey underscores the importance of continued dialogue and engagement with RO physicians as stakeholders in the ongoing efforts to reform payment structures within the specialty. Ultimately, the successful implementation of an EBP model will require careful consideration of the diverse needs and concerns of patients treated across the full spectrum of practices.

## Appendices

Appendix 1 (survey questions)

1. What best describes your experience level?

   o   Radiation oncologist 21 years or more out of training

   o   Radiation oncologist 11 - 20 years out of training

   o   Radiation oncologist 6 - 10 years out of training

   o   Radiation oncologist 5 years or less out of training

   o   Radiation oncology resident physician

   o   I am not a physician

2. What best describes your typical weekly work hours?

   o   Less than 30 hours

   o   30 - 39 hours

   o   40 - 49 hours

   o   50 - 59 hours

   o   60 - 69 hours

   o   70 - 79 hours

   o   At-least 80 hours

   o   Unemployed

   o   Retired

3. Which best describes your practice size? Count all radiation oncologists associated with your practice, not just those who practice at the same site as you.

   o   1 radiation oncologist (i.e., solo practice)

   o   2-5 radiation oncologists

   o   6-10 radiation oncologists

   o   11-19 radiation oncologists

   o   20 or more radiation oncologists

   o   Not currently practicing radiation oncology

4. Which best describes your primary practice type?

   o   Academic practice (including primary and satellite clinics)

   o   Single-specialty community or private practice

   o   Multi-specialty community or private practice

   o   Veterans Health Administration

   o   Military Treatment Facility

   o   Not currently practicing radiation oncology

5. What best describes your primary practice site?

   o   Freestanding Radiation Therapy Center (i.e., office)

   o   Hospital Outpatient Department

   o   Hospital Inpatient Department (i.e., treating admitted patients)

   o   Veterans Health Administration

   o   Military Treatment Facility

   o   Not currently practicing radiation oncology

6. What best describes your practice setting?

   o   Urban

   o   Suburban

   o   Rural

7. Where do you primarily practice?

8. Does your practice treat patients with real-time adaptive radiotherapy?

9. Does your practice treat patients with proton beam therapy?

10. Does your practice treat patients with brachytherapy?

11. Does your practice treat patients with radiopharmaceuticals?

12. Do you practice at a Prospective Payment System (PPS) Exempt Cancer Hospital?

13. Is your practice accredited by a national credentialing body?

14. Which of the following scenarios applies to you? Please check all that apply.

   o   I work locum tenens

   o   I am paid a predetermined base salary

   o   A component of my base salary is specifically allocated for administrative tasks

   o   I am eligible for a productivity bonus if I exceed a target number of work relative value units (wRVUs)

   o   I receive compensation directly from professional component (PC) payments

   o   I receive compensation directly from technical component (TC) payments

15. I feel confident in my ability to answer questions about current and proposed Medicare payment models for radiation oncologists.

16. I support annual adjustments to radiation oncology payments to account for inflation.

17. I support site-neutral payments that equalize compensation for radiation oncology services regardless of whether treatments are delivered at a freestanding radiation therapy center or hospital outpatient department.

18. I support paying radiation oncology practices for providing transportation services to patients who do not have reliable transportation to their treatments.

19. I support adjusting radiation oncology payments based on whether a practice is accredited by a recognized credentialing body.

20. Episode-based payments for radiation oncology services in which the payment amount is primarily based on the site of disease being treated and not on the x-ray beam modality or fraction number would result in more simplified billing.

21. Episode-based payments for radiation oncology services in which the payment amount is primarily based on the site of disease being treated and not on the x-ray beam modality or fraction number would result in more stable and predictable payments.

22. Episode-based payments for radiation oncology services in which the payment amount is primarily based on the site of disease being treated and not on the x-ray beam modality or fraction number would result in better alignment of financial incentives with clinical guidelines.

23. If episode-based payments for radiation oncology services are implemented, real-time adaptive radiotherapy should be excluded from that model and continue to be paid for through the current fee-for-service system.

24. If episode-based payments for radiation oncology services are implemented, proton beam therapy should be excluded from that model and continue to be paid for through the current fee-for-service system.

25. If episode-based payments for radiation oncology services are implemented, brachytherapy should be excluded from that model and continue to be paid for through the current fee-for-service system.

26. If episode-based payments for radiation oncology services are implemented, radiopharmaceuticals should be excluded from that model and continue to be paid for through the current fee-for-service system.

27. If most radiation oncologists support a specific episode-based payment model, then I would be willing to support a 1% reduction in Medicare payments for radiation oncology services in exchange for federal legislation that implements that model.

28. I support the implementation of episode-based payments for radiation oncology services in which the payment amount is primarily based on the site of disease being treated and not on the x-ray beam modality or fraction number.

29. I support site-neutral direct supervision requirements with limited exceptions (e.g., allow for tumor board attendance, allow for completion of emergency department and inpatient consultations).
